# Magnitude of common mental disorders and associated factors among patients with Epilepsy in Amhara regional state, Northwest Ethiopia

**DOI:** 10.1186/s12888-022-04314-2

**Published:** 2022-11-01

**Authors:** Getasew Mulat Bantie, Ashenafi Abate Woya, Girum Meseret Ayenew, Agumas Fentahun Ayalew, Abraham Amsalu Berneh

**Affiliations:** 1 Amhara National Regional State Public Health Institute, Bahir Dar City, Ethiopia; 2 Alkan Health Science Business and Technology College, Bahir Dar City, Ethiopia; 3 Department of statistics, Bahir Dar University, Bahir Dar, Ethiopia; 4 Department of Nutrition and Dietetics, School of Public Health, College of Medicine and Health Sciences, Bahir Dar University, Bahir Dar, Ethiopia; 5 College of Health Science, Department of Epidemiology, Injibara University, Injibara, Ethiopia

**Keywords:** Fenote Selam, Screening, Mental disorder, Patients with Epilepsy, Ethiopia

## Abstract

**Background:**

Common mental disorders are severe and frequent co-morbid psychiatric illnesses with epilepsy. Different study findings across the world showed that patients with epilepsy have a higher burden of mental disorders than the general population. However, these issues in patients with epilepsy have been consistently undiagnosed.

**Objectives:**

The study aimed to screen common mental disorders and the determinants among patients with epilepsy attending at Fenote Selam hospital.

**Methods:**

An institutional-based cross-sectional study was conducted among patients with Epilepsy from March 10 to May 15, 2019. Patients were assessed for the risk of common mental disorders using a pretested, structured, self-reporting questionnaire (SRQ-20). The collected data were entered into Epi-data version 3.1 software and analyzed using R version 4.0 software. Descriptive statistics were computed using frequency, percent, mean, and standard deviations. A simple logistic regression model was fit to identify the association and strength of exploratory variables and common mental disorders at a 95% confidence interval and p-value < 0.05.

**Results:**

The study included 202 patients diagnosed with epilepsy and yielded a response of 91.4%. About 53% of the patients were males. The magnitude of common mental disorders among patients with epilepsy was 57.9% (95% CI: 44.56, 71.24). Being more than one substance user (AOR = 5.7; 95%CI: 1.6, 20.7) and Not having social support (AOR = 4.3; 95%CI: 1.5, 11.9) were the identified determinants of common mental disorders.

**Conclusion:**

The magnitude of common mental disorders among patients with epilepsy were high. Not having social support and khat chewing were the identified risk factors significantly associated with common mental disorders. Early screening and treatments are the key interventions to prevent complications and deaths from common mental disorders.

## Background

Mental health is the crucial aspect of health closely allied with the physical and physiological changing aspects of the human body [1]. A clinically important impairement in a person’s cognition, emotion control, or behavior caused by a failure in the biological, psychological or developmental processes underpinning mental functioning is known as a mental disorder [2]. As well as insomnia, fatigue, irritability, forgetfulness, difficulty concentrating, substantial distress, impairment of social, or occupational activities, and somatic complaints, mental disorders encompass a wide range of mental disorders that do not fit into standard diagnostic criteria [3–5].

The World Health Organization reported that mental disorder is the direct risk factor for mortality and morbidity [6, 7]. Psychiatric comorbidities occur in about one-third of patients with epilepsy during the lifespan, and the risk of these comorbidities are much higher in patients with treatment-resistant seizures [8, 9].

Mental disorders have a vital negative impact on the quality of life and living standards of patients with epilepsy. Risk factors for common mental disorders among epileptic patients were being female, young age, marital status, lower-income, unemployment, low educational status, worse QOLIE-89 scores, lack of social support, frequent seizures attacks, side effects of antiepileptic drugs, medication non-adherence, nicotine dependence, alcohol misuse, family history of psychiatric illness, comorbidity of medical condition, duration of illness, and poly-pharmacy [10–16].

Despite the high burden of common mental disorders among patients with epilepsy, they remain under-investigated and inappropriately treated [18]. Better understanding and treatment of common mental disorders can assist early complication management and better health outcomes for people living with epilepsy. However, the magnitude and determinants of common mental disorders in patients with epilepsy are not determined well in low income countries, including Ethiopia in general and the study area in particular. Therefore, this study aimed to assess the magnitude of common mental disorders and the determinants among patients with epilepsy attending at the Fenote Selam hospital.

## Methods

### Study design, setting, and period

This institutional-based cross-sectional study was conducted at the Fenote Selam hospital from March 10 to May 15, 2019. The hospital is situated 378 km from Ethiopia’s capital city of Addis Ababa in the Amhara region. It is the only public hospital in Fenote Selam town.

### Population

The source population for this study was all patients with epilepsy (N = 521) attending at the Fenote Selam hospital for epilepsy treatment, while the study population was those patients 18 years and older. Those patients who dropped out the treatment, missed the appointment, or transferred out to other health institutions during the data collection period were excluded.

### Sample size determination and sampling procedure

The sample size was determined using the single population proportion formula by considering a 95% confidence level, a 5% margin of error, and a 50% proportion of common mental disorders. Taking the 10% non-response rate and the correction formula (N < 10, 000), the final sample size was 202. A simple random sampling technique was applied using their medical record numbers. The patients were interviewed and their medical records reviewed.

### Operational definitions

#### Common Mental disorder

The patient was screened for common mental disorder using the SRQ-20 dichotomous items (Yes = 1, No = 0). The likelihood of a common mental disorder was considered when he/she responded to nine or more positive (yes) answers out of the total. Otherwise, the patient was considered not at risk of common mental disorder [19].

#### Social support

Patients with Epilepsy who scored mean or above of the social support assessing questions (got counseling, financial aid, and or physical support from family, friends) correctly were considered as getting social support. Otherwise didn’t get social support [20].

### Data collection tool

Data were collected using a pre-tested structured questionnaire, adapted from a standardized self-reporting questionnaire (SRQ-20) with a 30-day recall period [21, 22]. The questionnaire comprised social-demographic, somatic, depressive/anxiety, and cognitive [23] and the multidimensional scale of perceived social support [20] assessing characteristics. It was translated into the Amharic (the indigenous) language by the independent translator (Ph.D. in linguistics) and then back to English to check for consistency. The five-day training was given to two enumerators (BSc in nursing) and one supervisor (MSc in psychiatry). The enumerators conducted a role-plays before the actual data collection period. Finally, the data was collected using the Amharic version of the questionnaire. Each questionnaire was examined for completeness and consistency by the supervisor and the principal investigator daily, and appropriate feedback was given to the data collectors.

### Data management and analysis

Data entry, cleaning, and coding were performed using Epi-data version 3.1 software, and the analysis was done using R version 4.0 software. Descriptive statistics were computed using frequency, percent, mean, and standard deviation. Bivariate and multivariate logistic regression analyses were employed to assess the association between the exploratory variables and mental disorders. The strength of the association was measured using the adjusted odds ratio (AOR) and 95% confidence interval (CI). A p-value < 0.05 was considered a statistically significant predictor of common mental disorders.

## Results

### Socio-demographic characteristics of the study participants

A total of 202 patients with epilepsy participated in the study, with a response rate of 91.4%. About 52% and 54% of the patients were males and single. About 20% of the patients were unable to read and write. About one-third of the patients were students. Almost 40% of them were living in rural areas and had a monthly income of fewer than 700 Birrs **(**Table 1**).**

**Table 1:** Socio-demographic characteristics of the respondents of Fenote Selam hospital, northwest Ethiopia, 2019

Variable	Category	Frequency	Percent
Sex	Male	106	52.5
	Female	96	47.5
Age (in years)	18–24	110	54.5
	25–34	49	24.3
	35–44	18	8.9
	45^+^	25	12.3
Marital Status	Single	109	54.0
	Married	72	35.6
	Divorced	21	10.4
Educational Status	Unable to read and write	40	19.8
	Able to read and write only	32	15.8
	Primary	68	33.7
	Secondary	43	21.3
	College graduate	19	9.4
Occupational Status	Farmer	46	22.8
	Government employee	14	6.9
	Self-employee	52	25.7
	Student	70	34.7
	Day laborer	20	9.9
Religion	Orthodox	120	59.4
	Protestant	60	29.7
	Muslim	22	10.9
Residence	Urban	121	59.9
	Rural	81	40.1
Monthly income (in Birr)	< 700	81	40.1
	700–1499	79	39.1
	≥ 1500	42	20.8

### Seizure characteristics of patients with epilepsy

About 52% of the current and ever substance-using patients were at risk of common mental disorders. Of 35.3% of multi-substance users, 72% were at risk of common mental disorders. Of 20.3% of phenytoin drug users, 76% were at risk of common mental disorders. Similarly, out of 20.3% of patients who had a seizure during treatment, 76% were at risk of having a common mental disorders **(**Table 2**).**

**Table 2:** Seizure related characteristics of the respondents at the Fenote Selam hospital, northwest Ethiopia, 2019

Variable	Category	Common mental disorders	
		**Yes** **N (%)**	**No** **N (%)**
Used addiction causing-substances	Yes	53 (45.3)	49 (54.7)
	No	64 (54.7)	36 (45.3)
Substance user	Current user	23 (52.3)	21 (47.7)
	Ever user	30 (51.7)	28 (48.3)
Types of substances used	More than one user	26 (72.2)	10 (27.8)
	Alcohol	6 (25)	18 (75)
	Tobacco	12 (63.2)	7 (36.8)
	Chat	9 (39.1)	14 (60.9)
Types of anti-epileptic drug used	Phenobarbital	84 (56.7)	64 (43.3)
	Phenytoin	31 (75.6)	10 (24.4)
	Combined drugs	2 (15.4)	11 (84.6)
Duration of epilepsy	≤ 2 years	84 (58.7)	59 (41.3)
	3–5 years	17 (68.0)	8 (32.0)
	> 5 years	16 (47.1)	18 (52.9)
Social support	Yes	62 (42.0)	67 (58.0)
	No	23 (31.5)	50 (68.5)
Seizure during treatment	Yes	31 (75.6)	10 (24.4)
	No	86 (53.4)	75 (46.6)
The frequency of seizures in the last month	One	15 (48.4)	5 (51.6)
	Two and above	16 (51.6)	5 (48.4)

### The magnitude of common mental disorders among patients with epilepsy

The magnitude of common mental disorders among patients with epilepsy was 57.9% (95% CI: 44.56, 71.24).

### Factors associated with a diagnosable mental disorder

On bivariate analysis, occupational status, social support, types of substances used, types of anti-epileptic drugs used, and seizures during treatment were factors associated with common mental disorders at 20% of level of significance. Whereas in the multivariable analysis, only social support and types of substances used showed a significant association with common mental disorders.

For those patients who hadn’t received social support, the odds of developing common mental disorders were about four (AOR = 4. 3; 95%CI: 1.5, 11.9) times higher compared to those who had. Similarly, for those patients abused by more than one substance, the odds of common mental disorders were about six (AOR = 5. 7; 95%CI: 1.6, 20.7) times higher compared to those khat chewers **(**Table 3**)**.

**Table 3:** Factors associated with common mental disorders at the Fenote Selam hospital, northwest Ethiopia, 2019

Variable	Common Mental Disorder		COR (95%CI)	AOR (95%CI)	P-value
	**No (N)**	**Yes (N)**			
**Occupational status**					
Farmer	12	34	1.2 (0.4, 3.9)	1.45 (0.2, 14.1)	
Government employee	8	6	0.3 (0.8, 1.3)	0.23 (0.02, 3.2)	
Self-employed	20	32	0.7 (0.2, 2.1)	0.3 (0.03, 2.6)	
Student	39	31	**0.3 (0.12, 0.9)**	0.4 (0.04, 3.8)	0.432
Day laborer	6	14	1.00	1.00	
**Social support**					
Yes	62	67	1.00	1.00	
No	23	50	**2.1 (1.1, 3.7)**	**4.3 (1.5, 11.9)**	**0.006**
**Types of substances used**					
More than one substance user	10	26	**4.1 (1.3, 12.3)**	**5.7 (1.6, 20.7)**	**0.008**
Alcohol only	18	6	0.5 (0.2, 1.8)	0.4 (0.1, 1.8)	
Tobacco only	7	12	2.7 (0.8, 9.3)	3.6 (0.8, 15.8)	
Khat only	14	9	1.00	1.00	
**Types of anti-epileptic drugs used**					
Phenobarbital	64	84	1.00	1.00	
Phenytoin	10	31	**2.4 (1.1, 5.2)**	0.9 (0.3, 3.5)	
Combined drugs	11	2	**0.1 (0.03, 0.7)**	0.2 (0.02, 1.9)	
**Seizure during treatment**					
Yes	10	31	**2.7 (1.2, 5.9)**	0.2 (0.7, 1.7)	0.207
No	75	86	1.00	1.00	

## Discussion

This study identified the magnitude of common mental disorders and the determinants among patients with epilepsy attending the Fenote Selam hospital. The study revealed that 57.9% (95% CI: 44.56, 71.24) of the patients had common mental disorders. This finding was higher than the studies done in northwest Ethiopia (45.2%), Addis Ababa, Ethiopia (27.1%), Mexico (36.6%), Sudan (45.5%), the systematic review (32.71%), Hawassa, Ethiopia (34.2%), and Nigeria (31–37%) [17, 24–29], respectively. However, it was lower compared to the studies from Burkina Faso (67.3%) and Nigeria (37%) [30, 31]. The possible reasons for the difference might be due to differences in the study period, study area, and socio-cultural practice.

The present study showed that patients who had no social support were about fourfold more likely to have common mental disorders (AOR = 4. 3; 95%CI: 1.5, 11.9) compared to those who had social support. This finding was consistent with a study done in Ethiopia [27]. This could be explained by the fact that lack of social support might deteriorate the patient’s health and quality of life due to fear of lacking support in the future when he/she gets sick.

The study also identified the use of addiction-inducing substances while on anti-epilepsy treatment was significantly associated with common mental disorders. For patients who used more than one substance, the odds of common mental disorders (AOR = 5. 7; 95%CI: 1.6, 20.7) were 6 times higher compared to those who used chat. This was supported by a study done in central Ethiopia [32]. The possible justification for this typical finding might be that the content of chemicals in more substance users might bring a higher risk of common mental disorders than only chat users when taken along with anti-epileptic drugs.

The findings of this study indicate that common mental disorders are common co-morbidities in patients with epilepsy. Healthcare professionals should assess and treat psychiatric and physical co-morbidities among patients with a history of seizures to improve patient health outcomes. The families of patients with epilepsy should be made aware of the disorders and their related psychological co-morbidities so that the patients can receive sufficient support from their families.

### Limitations of the study

The difficulty of distinguishing the temporal relationships. There might also be a potential for recall bias. It is difficult to investigate why the effect of khat chewing is a potential risk factor for common mental disorders. Therefore, a strong evidenced study should be done to ascertain the impact of khat on mental disorders.

## Conclusion

The risk of common mental disorders among patients with epilepsy was high. Social support and the type of substances used were the identified factors significantly associated with the risk of common mental disorders.

## Recommendations

The authors recommended that the health care workers should give great attention to counseling on the reduction of substance use of the patients with epilepsy. In addition, the study suggests arranging social support and creating awareness about the consquences of taking substances (non medication) are important to reduce the common mental disorders.

## Data Availability

All data generated or analyzed during this study are included in this published article.

## Abbreviations

AOR: Adjusted Odds Ratio
BSc: Bachelor in Science
CI: Confidential Interval
COR: Crude Odds Ratio
Epi-data: Epidemiological Data
Ph.D: Doctor of Philosophy
SRQ: Self-Reporting Questionnaire
MSc: Masters of Science
